# Polycomb/Trithorax group proteins collaborate with Heterochromatin protein 1 to regulate *Drosophila* sex determination

**DOI:** 10.1186/1756-8935-6-S1-P70

**Published:** 2013-03-18

**Authors:** Janel Rodriguez, Jamila I Horabin

**Affiliations:** 1 Biomedical Science, Florida State University, Tallahassee, Florida, 32306, USA

## Background

A combination of histone modifications, a ‘histone epigenetic code’, is created and altered by specific enzymes, and recognized by proteins that bind to these modifications through specific domains [1]. These proteins modify the chromatin environment for precise transcriptional regulation. Some of these well studied chromatin remodeling complexes include the Polycomb and the Trithorax Group Proteins (PcG and Trx-G). The two groups are primarily antagonistic and control patterning of the body during embryogenesis, through the regulation of gene expression. The Polycomb Repressive Complexes mediate the initiation of gene repression and its maintenance. The Trx-G proteins are involved in maintaining the activation of gene expression [1]. In *Drosophila*, one of the earliest developmental decisions made in the embryo is that of determining its sex. This process involves regulating the expression of the X chromosome sensing promoter of *Sex-lethal* (*Sxl*) at its establishment promoter, *Sxl_pe_*[2]. Using this sensitive system which differentiates one versus two X chromosomes, our lab has shown that heterochromatin proteins are required for proper *Sxl_pe_* regulation. We found that Heterochromatin Protein 1 a (HP1a) plays both a repressive and activating role in regulating *Sxl_pe_*[3].

## Materials and methods

*Drosophila* Stocks: ash1[MB03235]/TM6C, Sb; E(z)[EY21318]; E(z)32A40/TM6C, Sb, Tb; Su(z)12[4]/TM6C, Sb, Tb; wild-type w[1118] and Ore R. *In situ* hybridization in 0-4 hour embryos was used to analyze *Sxl_pe_* expression levels using a Digoxygenin labeled RNA probe specific to *Sxl_pe_* transcripts; Chromatin Immunoprecipitations were performed with antibodies specific to H3K4me3, H3K27me3 and HP1a on chromatin prepared from 1-3 and 2-4 hour embryos, also from the above mentioned stocks. Quantitative real-time PCR quantified *Sxl* sequences in the DNA from each ChIP.

## Results

We find that the PcG/Trx-G proteins genetically interact with mutations in the sex determination pathway and influence the ability of females to determine their sex. In situ analysis of a*sh1*, *Su*(*z*)*12* and *E*(*z*) mutant embryos show they affect both the timing and strength of transcription of the sex establishment promoter, *Sxl_pe_.* qRT-PCR analyses support the in situs, showing a change in Sxl_Pe_ mRNA levels. We also observe that these proteins are necessary for proper histone 3 lysine 4 (H3K4) and histone 3 lysine 27 (H3K27) methylation at the promoter. Surprisingly, we find that embryos deficient in E(z), Su(z)12 or ASH1 protein also affect the binding of HP1a at *Sxl_Pe_* sequences.

## Conclusions

Our data support the idea that PcG proteins function on a larger set of target genes with a broader role in gene regulation and development. Additionally, we find a novel heterochromatin role for PcG/Trx-G proteins which influences HP1a in the regulation of gene expression.
